# Abnormal Cortical Thickness Development in Young Adults With Heavy Cannabis Use: A Longitudinal Study

**DOI:** 10.1111/adb.70040

**Published:** 2025-05-08

**Authors:** Wei Li, Cheng Xu, Hanyuan Xu, Bo Yin, Hui Xu, Dandong Li

**Affiliations:** ^1^ Department of Neurosurgery The Second Affiliated Hospital and Yuying Children's Hospital of Wenzhou Medical University Wenzhou China; ^2^ School of Mental Health Wenzhou Medical University Wenzhou China; ^3^ Alberta Institute, Wenzhou Medical University Wenzhou China

**Keywords:** cortical thickness, heavy cannabis use, longitudinal design, orbitofrontal cortex

## Abstract

Cannabis is one of the most commonly used illicit drugs worldwide, with its prolonged use potentially leading to various cognitive impairments and brain structural changes. However, current research on the dynamic changes in cortical thickness (CT) related to cannabis use remains limited, especially regarding the relationship between the severity of cannabis use and CT changes in heavy cannabis use (HCU). This study employed a longitudinal design to investigate CT changes in young adults with HCU from baseline (BL) to 3‐year follow‐up (FU). The results showed a significant group effect in the left lateral orbitofrontal cortex (OFC), and a significant time effect revealed CT changes in several brain regions, including the left lateral frontal cortex, bilateral medial frontal cortex, bilateral posterior cingulate cortex and bilateral insula. Simple effects analysis further demonstrated that the CT of left lateral OFC in young adults with HCU decreased significantly at FU compared with their BL and was also lower than control group at FU. Furthermore, a significant positive correlation was observed between the total score of Cannabis Use Disorders Identification Test at FU and the CT of left lateral OFC. These findings suggest that prolonged cannabis use may disrupt the structural integrity of the left lateral OFC, impairing decision‐making, impulse control and emotional processing, thereby exacerbating addictive behaviours. This study provides key evidence for understanding the neural mechanisms underlying cannabis addiction.

## Graphical abstract

At BL, there was no significant difference in the CT of the left lateral OFC between the two groups. However, at FU, young adults with HCU exhibited significantly lower CT in the left OFC compared to their own BL and to the HC group at the same time point. Moreover, at FU, there was a significant correlation between the CUDIT total score and the CT of the left lateral OFC.

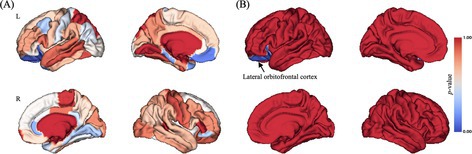

## 1 Introduction

Cannabis is the most commonly used illicit drug worldwide, with an estimated 219 million users in 2021 [1]. Cannabis use is particularly prevalent among young people. The 2022 National Survey on Drug Use and Health in the United States show that approximately 22% of people aged 12 or older (61.9 million people) reported using cannabis in the past year. Among young people aged 18 to 25, the proportion is significantly higher, nearly 38.2% (13.3 million people) [2]. Previous studies consistently highlight that heavy cannabis use (HCU) is associated with a variety of adverse effects, including increased impulsivity, impaired decision‐making, lack of pleasure, increased anxiety and depression and an increased risk of psychotic symptoms [3, 4, 5]. In addition, rising cannabis use has been linked to increased engagement in risky behaviours, higher crime rates and a heavier burden on healthcare systems, which has increased social costs [5]. The legalization of cannabis in several countries and regions has further lowered the threshold for cannabis use, prompting global concerns about its addictive nature and the resulting personal and social consequences [6].

Advances in neuroimaging techniques have played a key role in exploring how cannabis use affects brain structure. Magnetic resonance imaging (MRI) studies have consistently reported structural and functional abnormalities in amygdala, prefrontal cortex and inferior parietal lobule following HCU, which responsible for emotion regulation, cognitive control and memory processes [7, 8]. Previous research has shown that cannabis use disrupts the activation patterns of these regions to cannabis‐related cues, impairs functional connectivity between the amygdala and dorsolateral prefrontal cortex and compromises emotion regulation and decision‐making [8, 9]. In addition, HCU has been associated with lower grey matter volume (GMV) of hippocampal, thalamic and prefrontal cortex, which mirrors deficits in learning, memory and reward‐related systems in young adults [10, 11]. Cannabis‐related changes in amygdala volume have also been observed [12, 13].

However, cortical thickness (CT) provides valuable insights into changes in brain microstructure. Unlike GMV, CT provides information on different genetic and cellular processes, offering a more region‐specific perspective on brain changes induced by drug use [14, 15]. Previous studies have reported lower CT of the right superior temporal sulcus, anterior cingulate cortex and cingulate gyrus and left superior temporal sulcus and anterior central gyrus in chronic cannabis users [16]. Similarly, lower CT in the medial temporal lobe, orbitofrontal cortex (OFC) and cingulate regions were found in young adults with HCU, and these reductions were associated with impaired performance of verbal learning [17]. However, longitudinal studies have produced mixed results. One study of early adolescence reported that increased cumulative exposure to cannabis was associated with increased CT in frontal and parietal regions [18], while a large cohort study based on IMAGEN data found a significant negative correlation between five‐year cumulative cannabis use and prefrontal CT in adolescents who started using cannabis at an average age of 14 years [19]. Despite these discrepancies, existing studies largely agree that cannabis use leads to alterations in CT, particularly in regions critical for emotion regulation, impulse and cognitive control [12, 16, 19]. However, most studies rely on cross‐sectional designs, which limits the ability to determine whether structural abnormalities are a direct consequence of cannabis use or a pre‐existing vulnerability.

To address these gaps, the current study used surface‐based morphometry to examine the dynamic effects of HCU on CT over 3 years. Specifically, the study aimed to (1) assess CT changes at baseline (BL) and follow‐up (FU) in young adults with HCU compared with healthy controls (HC); and (2) explore the relationship between CT and cannabis use severity at BL and FU. We hypothesized that (1) there were significant group‐by‐time interaction effect in the left lateral orbitofrontal cortex (OFC), and the CT of left lateral OFC in young adults with HCU decreased significantly at FU compared with their BL and was also lower than control group at FU; (2) there was a significant correlation between CT of left lateral OFC and cannabis use severity in young adults with HCU.

## 2 Methods

### 2.1 Participants

For this study, we utilized data obtained from the OpenNEURO database (https://openneuro.org/datasets/ds000174/versions/1.0.1). Our analysis included 20 young adults with HCU and 22 matched noncannabis use healthy controls (HC). All participants underwent MRI scanning and clinical assessments at BL and FU stages, with an average interval duration of 39 ± 2.4 months. At the BL, HCU was defined as the consumption of cannabis for more than 10 days per month consistently for a minimum of 2 years without seeking treatment or having a history of cannabis treatment [20]. HC in this study were individuals who had used cannabis fewer than 30 times in their lifetime and had not used it within the past year [20]. Detailed information regarding the informed consent process with all participants could be found in previous research [20].

### 2.2 Clinical Assessments

The severity of cannabis use was assessed using the Cannabis Use Disorder Identification Test (CUDIT) [21]. In addition, the severity of alcohol use was assessed using the Alcohol Use Disorder Identification Test (AUDIT) [22]. Additionally, the Mini International Neuropsychiatric Interview [23] was conducted by two experienced psychologists blinded to the study to assess the prevalence of mental disorders. Detailed information regarding participant characteristics and clinical evaluations could be found in previous research [20].

### 2.3 MRI Data Acquisition

MRI scanning was conducted using a Philips Healthcare 3.0T MRI scanner. Participants' heads were secured in position using a custom‐built head holder during the scanning process to ensure stability and accuracy. High‐resolution structural images were acquired with the following parameters: echo time = 4.16 s, repetition time = 9.6 s, flip angle = 8°, slice thickness = 1.2 mm, field of view = 256 mm × 256 mm, matrix size = 256 × 256, voxel size = 1 × 1 × 1.2 mm^3^ and 182 slices.

### 2.4 MRI Data Preprocessing and Measurement of CT

Each participant's structural T1‐weighted MRI data were preprocessed using FreeSurfer v7.2.0 software package (http://surfer.nmr.mgh.harvard.edu). For detailed information regarding the surface‐based morphology analysis, I recommend referring to the previous studies where the specifics of this analysis were documented [24, 25, 26]. The FreeSurfer pipeline processing involved several steps, including removal of nonbrain tissue, Talairach transformation, intensity normalization, grey/white matter boundary tessellation, topology correction, surface deformation, registration to a common spherical atlas and cortical surface reconstruction. To obtain measurements of CT, the cortical morphologies were smoothed using a 10‐mm full‐width‐at‐half‐maximum Gaussian kernel, following methodologies described in previous research [27, 28, 29]. CT was calculated at each vertex in the cortex by measuring the distance between the pial surface and the grey‐white matter surface. This approach provides a local assessment of CT across the entire cortical surface. All outputs underwent meticulous inspection throughout the preprocessing phase, and manual corrections were applied as necessary. Subsequently, the average values of CT within 34 cortical parcellations were determined in each hemisphere and defined by the Desikan atlas [30]. CT of each cortical region was exported for subsequent analysis.

### 2.5 Statistical Analysis

Demographic and clinical characteristics of all participants were analysed using R (Version 4.1.3; R Core Team [31]) and RStudio (‘Ghost Orchid’ Release; R Core Team [31]). Independent samples *t*‐tests were used to evaluate group differences in age at baseline and age at first cannabis use, while chi‐square tests were used to measure gender differences. For the score of CUDIT and AUDIT, and CT of all cortical regions, two‐way mixed ANOVA with a between‐subjects factor ‘group’ (young adults with HCU and HC) and a within‐subjects factor ‘time point’ (BL and FU) were performed. When the group‐by‐time point interaction was significant, simple effect analysis was performed, and *p* values were Bonferroni corrected. Then, a correlation analysis was conducted between total score of CUDIT and CT of left lateral orbitofrontal cortex at both BL and FU in young adults with heavy cannabis use. The significance threshold was set at *p* < 0.05 after Bonferroni corrected for multiple comparisons.

## 3 Results

### 3.1 Participants and Characteristics

No significant differences were found in age at BL (*t*(40) = −1.465, *p* = 0.151) and sex (*χ* ^2^_(1)_ = 0.213, *p* = 0.645) between young adults with HCU and controls. Young adults with HCU demonstrated a significantly earlier age at the onset of first cannabis use compared with controls (*t*(40) = −4.367, *p* < 0.001), and the mean age at onset of frequent cannabis use for young adults with HCU was 16.20 ± 2.38 years old. However, there was no significant ‘group’ × ‘time point’ interaction effect on the score of CUDIT (*F*_(1, 80)_ = 0.033, *p* = 0.855) and score of AUDIT (*F*_(1, 80)_ = 0.082, *p* = 0.776). In the present study, none of the participants exhibited a prevalence of mental disorders. The demographic information of all participants is depicted in Table S1.

### 3.2 Group Main Effect on CT

**FIGURE 1:**
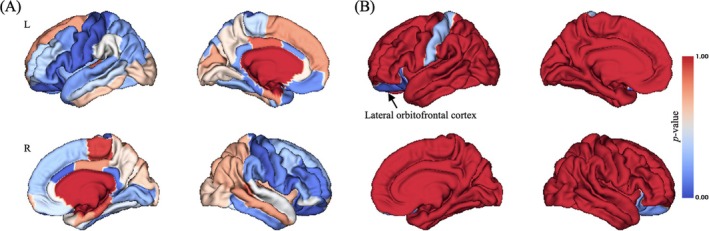
Brain pattern of group main effect on CT. (A) Uncorrected *p* value distribution for group main effect on CT; (B) Bonferroni correction of *p* value distribution for group main effect on CT. L, left; R, right. The colour bar represented *p* value of group main effect on CT.

While significant main effect of group on CT in widespread brain regions were observed (uncorrected *p* < 0.05; Figure 1A), only left lateral OFC still showed significant group main effect on CT after Bonferroni correction (Bonferroni corrected *p* < 0.001, *F* = 48.847; Figures 1B and 4A).

### 3.3 Time Point Main Effect on CT

**FIGURE 2:**
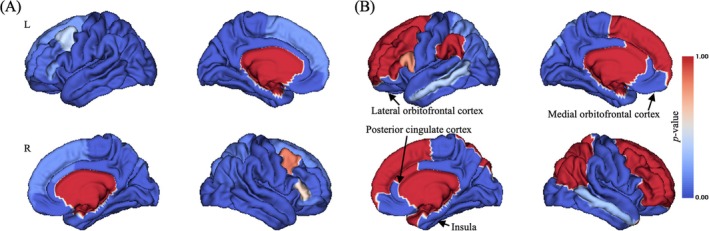
Brain pattern of time point main effect on CT. (A) Uncorrected *p* value distribution for time point main effect on CT; (B) Bonferroni correction of *p* value distribution for time point main effect on CT. L, left; R, right. The colour bar represented *p* value of time point main effect on CT.

Significant main effect of time point on CT in widespread brain regions were observed (uncorrected *p* < 0.05; Figure 2A), and there were still many brain regions exhibiting significant time point main effect on CT after Bonferroni correction (Bonferroni corrected *p* < 0.001; Figure 2B), including left lateral OFC (Figure 4B), bilateral medial orbitofrontal cortex, bilateral posterior cingulate cortex and bilateral insula (Figure S1).

### 3.4 Group‐by‐Time Point Interaction Effect on CT

**FIGURE 3:**
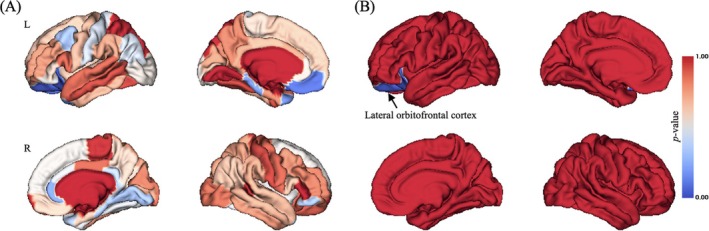
Brain pattern of group‐by‐time point interaction effect on CT. (A) Uncorrected *p* value distribution for group‐by‐time point interaction effect on CT; (B) Bonferroni correction of *p* value distribution for group‐by‐time point interaction effect on CT. L, left; R, right. The colour bar represented *p* value of group‐by‐time point interaction effect on CT.

**FIGURE 4:**
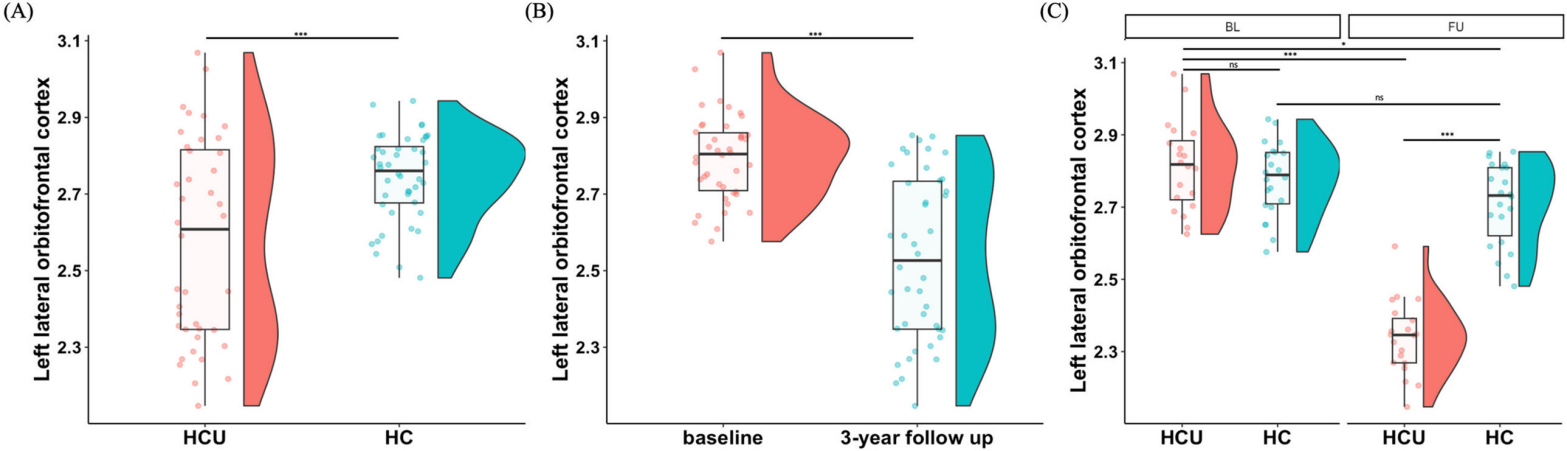
Simple effect analysis results for (A) group main effect; (B) time point main effect; (C) group‐by‐time point interaction effect of CT of left lateral orbitofrontal cortex. HCU, heavy cannabis use; HC, healthy control; BL, baseline; FU, 3‐year follow up. *, *p* < 0.05; ***, *p* < 0.001; ns, no significant.

While Significant interaction effect of group‐by‐time point on CT in limited brain regions were observed (uncorrected *p* < 0.05; Figure 3A), only left lateral OFC still showed significant group‐by‐time point interaction effect on CT after Bonferroni correction (Bonferroni corrected *p* < 0.001, *F* = 48.847; Figure 3B). Simple effect analysis found that at baseline, there was no significant difference of CT of left lateral OFC between groups (*p* > 0.05), but at 3‐year FU, young adults with HCU exhibited significant lower CT of left lateral OFC compared with HC (*p* < 0.001), as well as young adults with HCU at BL (*p* < 0.001; Figure 4C).

### 3.5 Correlation Analysis

**FIGURE 5:**
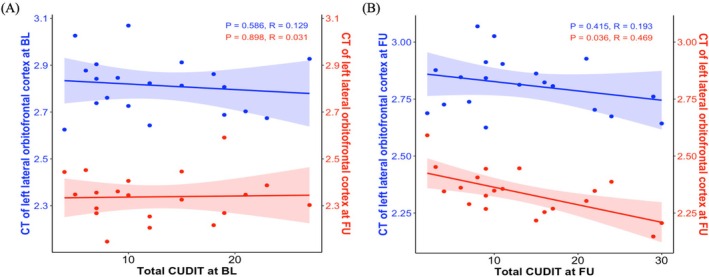
Correlation analysis results. (A) The relationship between total score of CUDIT at baseline and CT of left lateral orbitofrontal cortex at both and 3‐year follow up. (B) The relationship between total score of CUDIT at 3‐year follow up and CT of left lateral orbitofrontal cortex at both and 3‐year follow up. BL, baseline; FU, 3‐year follow up; CT, cortical thickness; CUDIT, Cannabis Use Disorder Identification Test.

At baseline, there were no significant associations between total score of CUDIT and CT of left lateral OFC at both baseline (*R* = 0.129, *p* = 0.586) and 3‐year follow up (*R* = 0.031, *p* = 0.898; Figure 5A). At 3‐year follow up, there was no significant association between total score of CUDIT and CT of left lateral OFC at BL (*R* = 0.193, *p* = 0.415), while significant correlation between total score of CUDIT and CT of left lateral OFCat 3‐year FU was observed (*R* = 0.469, *p* = 0.036; Figure 5B).

## 4 Discussion

This study utilized a longitudinal design to explore CT changes between young adults with HCU and HC at BL and 3‐year FU, as well as the association between CT and cannabis use severity. The results revealed a significant group effect in the left lateral OFC. Additionally, the time effect was significant in several regions, including the left lateral frontal cortex, bilateral medial frontal cortex, bilateral posterior cingulate cortex and bilateral insula. Simple effects analysis indicated that, at BL, there was no significant difference in the CT of the left lateral OFC between the two groups. However, at FU, young adults with HCU exhibited significantly lower CT in the left OFC compared with their own BL and to the HC group at the same time point. Moreover, at FU, there was a significant correlation between the CUDIT total score and the CT of the left lateral OFC.

The results showed that for the main effect of group, in the left lateral OFC, the group had a significant effect on CT. Specifically, compared with the HC, young adults with HCU had significantly less CT in the left lateral OFC. This further supports previous research [11]. As a core region that plays an important role in individual decision‐making and behavioural inhibition, the OFC is connected to subcortical regions such as the lateral amygdala and nucleus accumbens. By integrating information from these regions, the OFC can generate outcome expectations [32]. A decrease in CT in this region may mean that this prediction mechanism is impaired, making it difficult to incorporate previous negative outcomes triggered by cannabis use into the decision‐making process, and therefore continuing to use it regardless of the risks and consequences [33]. In addition, the OFC plays a key role in the integration and processing of sensory and emotional stimuli [34]. Some studies have pointed out that the levels of depression and anxiety in cannabis users are related to the functional connectivity between the left OFC and other brain regions [35]. The decrease in the CT of the left lateral OFC in the HCU group may also affect an individual's ability to cope with negative emotions and increase the risk of cannabis dependence.

The significant time effect observed in several brain regions (including the left frontal cortex, bilateral medial frontal cortex, bilateral posterior cingulate cortex and bilateral insula) highlights the dynamic nature of cortical thickness changes over time. Studies have shown that CT remains dynamic throughout the life course, reflecting processes such as myelination, synaptic pruning and neuroplasticity [36]. In young adults, the observed reductions in CT in these regions during FU may be consistent with natural neurodevelopmental processes such as synaptic pruning and remyelination, which contribute to the optimization of neural networks, cognitive maturation and emotional regulation [19]. However, in young adults with HCU, these natural processes may be altered or interrupted by the neurotoxic effects of chronic cannabis use, further emphasizing the role of cannabis in influencing CT dynamics.

Simple effect analysis showed that although there was no significant difference in CT of the left OFC between the two groups at BL, the CT of the left lateral OFC in young adults with HCU was significantly lower at FU compared with BL and HC at the same time point. Long‐term cannabis use has been shown to have neurotoxic effects on the brain, damaging both its structure and function [37]. The main psychoactive ingredient in cannabis, delta‐9‐tetrahydrocannabinol (THC), acts on the endocannabinoid system and may cause functional and structural abnormalities by overactivating CB1 receptors, which are densely expressed in the prefrontal cortex (including the OFC) [37]. This overactivation can lead to synaptic activity disruption, neuroinflammation and oxidative stress, which can lead to neuronal death [38]. In addition, THC can induce microglial activation and promote the expression of proinflammatory cytokines, a process that may lead to persistent inflammatory responses in the brain, thereby exacerbating local structural damage. Long‐term neuroinflammation not only affects neuronal survival but may also interfere with synaptic remodelling and neural network integration, further damaging OFC functions that are closely related to decision‐making and impulse control. In addition, THC's regulation of multiple neurotransmitter systems may interfere with normal synaptic plasticity and neurodevelopmental processes, leading to changes in brain structure [39]. However, there were no significant differences in OFC CT between the two groups at BL, which may reflect the relatively short period of cannabis use among the young adults with HCU. Animal studies have shown that the duration of cannabis exposure is a key determinant of its neurotoxic effects on brain structure and at least 3 months of cannabinoid administration are required to produce neurotoxicity in juvenile rodents, which corresponds to 7–10 years of cannabis use in humans [40]. Therefore, the lack of significant differences at BL may indicate that the young adults with HCU in this study had not yet experienced the cumulative neurotoxic effects of cannabis use at this time.

The significant positive correlation observed between the total CUDIT score and the left lateral OFC CT at FU suggests a close link between the severity of cannabis use and the structural integrity of the OFC. As an area closely related to social behaviour, the OFC is critical for decision‐making, reward processing, impulse regulation and emotion control [41, 42]. Structural damage to the OFC may indicate that individuals have difficulty resisting cannabis‐related impulses, exhibit reduced self‐control when using cannabis and are more sensitive to the rewarding properties of cannabis‐related cues, leading to an increase in frequency of use. Furthermore, the lack of significant correlations between CUDIT scores and OFC CT at BL and between BL CT and FU CUDIT scores highlights the cumulative and progressive nature of the neurotoxic effects caused by cannabis. Previous studies have shown that during adolescence, the brain is in a critical stage of rapid maturation and reconstruction. This period is not only accompanied by a reduction in cortical volume and an adjustment in grey matter concentration, but also by a substantial reorganization of connectivity in the neural network, such as synaptic pruning and myelination. Because of this, the adolescent brain is particularly sensitive to external interference. Ingredients such as THC in marijuana may interfere with these normal neural development processes, induce neurotoxicity and lead to structural damage [18, 43]. In this study, the average late age of cannabis exposure among participants may have attenuated this early damage, which explains the lack of a significant correlation at BL. Another study has suggested that cumulative lifetime cannabis use is associated with structural brain changes at FU [18], further supporting the idea that the impact of cannabis on brain structure depends on the amount and duration of exposure [41].

This study used a longitudinal design to investigate CT changes in young adults with HCU and HC over a 3‐year period, as well as the association between CT and cannabis use severity. The results showed a significant decrease in the left lateral OFC CT in young adults with HCU, highlighting the potential long‐term adverse effects of HCU on decision‐making, impulse control and emotional processing. In addition, the structural state of the left OFC may, to some extent, reflect the cumulative effects of cannabis use and the interference with decision‐making and impulse control mechanisms. This suggests that the cortical thickness of the left OFC may serve as a potential biomarker to help assess the severity of cannabis addiction. These functional impairments may further exacerbate addictive behaviours. This study provides new perspectives for understanding the neural mechanisms of cannabis addiction and offers potential targets for intervention strategies.

Despite its significant contributions, the study has several limitations. First, the small sample size of 20 young adults with HCU and 22 HC limits the generalizability of the findings. Future studies should include larger samples to validate the results. Second, the study did not explore individual differences such as gender, genetic predisposition or psychiatric comorbidity, which may influence the effect of cannabis use on brain structural changes. Third, the study focused on structural changes in CT but did not integrate multimodal imaging techniques such as functional MRI, which could reveal structural and functional abnormalities associated with HCU. Future studies should consider these factors and extend the follow‐up period to better capture the long‐term effects of HCU on brain structure and function. In addition, although no significant structural differences were found between the two groups at baseline, this may reflect that the initial cannabis use did not reach the threshold for cumulative neurotoxic effects or was affected by pre‐existing vulnerability factors. Due to the limitations of the study design, it is impossible to fully distinguish the relative contribution of cumulative cannabis exposure and innate susceptibility to brain structural changes. Future studies need to integrate more multimodal data to explore this relationship.

## 5 Conclusion

Young adults with CUD exhibited altered CT of the left lateral OFC, which was significantly positive correlated with cannabis use severity. These findings suggest that prolonged cannabis use may disrupt the structural integrity of the left lateral OFC, impairing decision‐making and impulse control, thereby exacerbating addictive behaviours.

## Author Contributions

**Wei Li**: conceptualization; methodology; data curation; formal analysis; investigation; writing – original draft. **Cheng Xu**: conceptualization; methodology; data curation; formal analysis; software; visualization; investigation; writing – original draft. **Hanyuan Xu**: conceptualization; methodology; data curation; formal analysis; software; visualization; investigation; writing – original draft. **Bo Yin**: funding acquisition; conceptualization; supervision; writing – review and editing. **Hui Xu**: funding acquisition; conceptualization; methodology; data curation; formal analysis; software; visualization; investigation; supervision; writing – review and editing. **Dandong Li**: funding acquisition; conceptualization; methodology; data curation; formal analysis; software; visualization; investigation; supervision; writing – review and editing.

## Ethics Statement

This study was approved and consented by the Ethics Committee of Wenzhou Medical University.

## Consent

The authors have nothing to report.

## Conflicts of Interest

The authors declare no conflicts of interest.

## Supporting information


**Table S1.** Demographic information of young adults with HCU and Controls.
**Figure S1.** Significant main effect of time point on CT in widespread brain regions.

## Data Availability

The data that support the findings of this study are available on request from the corresponding author. The data are not publicly available due to privacy or ethical restrictions.
