# Supplementary material for: Abnormal Cortical Thickness Development in Young Adults With Heavy Cannabis Use: A Longitudinal Study
Source: Addict Biol. 2025 May 8;30(5):e70040. doi: 10.1111/adb.70040 (PMC12061635; doi:10.1111/adb.70040)

**Supplementary materials**

| **Table S1**. Demographic information of young adults with HCU and Controls. | | | | | | |
| --- | --- | --- | --- | --- | --- | --- |
|  | Young adults with HCU | | Controls | | *t/χ^2^*-value | *P*-value |
| Age at baseline (years) | 20.53(2.11) | | 21.56(2.45) | | -1.465 | 0.151 |
| Gender (F/M) | 5/15 | | 8/14 | | 0.213 | 0.645 |
| Age at onset first cannabis use (years) | 14.50(1.65) | | 18.46(2.99) | | -4.367 | 0.001 |
| Age at onset frequent cannabis use (years) | 16.20(2.38) | | NA | | NA | NA |
| Time point | BL | FU | BL | FU | *F*-value | *P*-value |
| Score of CUDIT | 12.70(6.59) | 13.25(8.31) | 0.05(0.21) | 0.18(0.39) | 0.033 | 0.855 |
| Score of AUDIT | 6.25(3.35) | 8.50(4.96) | 4.41(3.38) | 6.18(3.45) | 0.082 | 0.776 |
| *Notes*. HCU, heavy cannabis use; CUDIT, Cannabis Use Disorder Identification Test; AUDIT, Alcohol Use Disorder Identification Test; NA, Not Available; BL, baseline; FU, follow-up. Data was presented as Mean (SD) unless otherwise stated | | | | | | |

**Figure S1.** Significant main effect of time point on CT in widespread brain regions


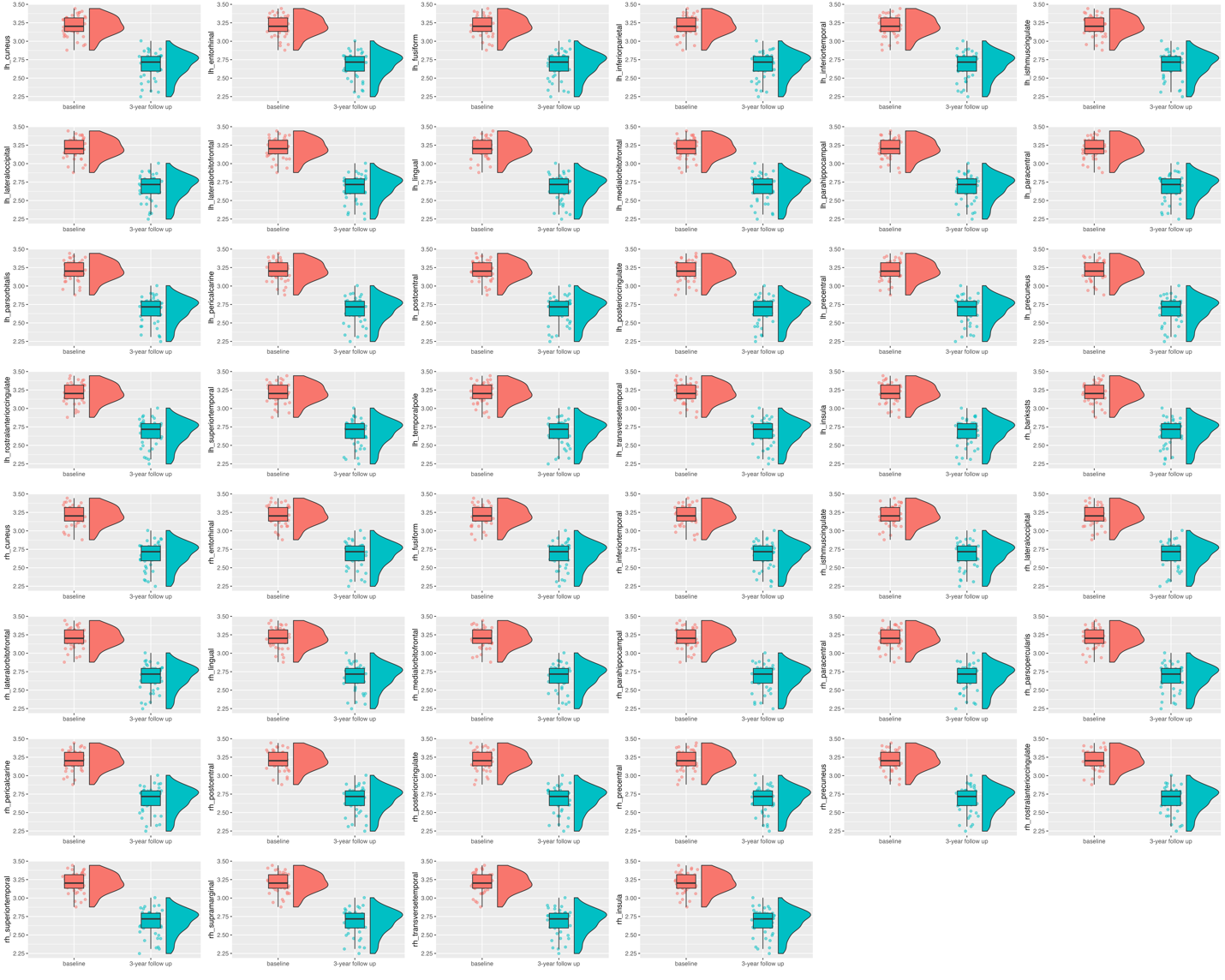

Supplement: Supplementary file 1 — Table S1. Demographic information of young adults with HCU and Controls. Figure S1. Significant main effect of time point on CT in widespread brain regions. [file ADB-30-e70040-s001.docx]
